# High-Gravity Brewing, Yeast Strain Selection, and Glucose Oxidase Effects on the Quality of Nonalcoholic Beer

**DOI:** 10.1021/acsomega.5c13295

**Published:** 2026-03-03

**Authors:** Christian Schubert, Kain Escobar, Rahul Sen, Dawn Maskell, Scott Lafontaine

**Affiliations:** 1 Department of Food Science, 3341 University of Arkansas, 2650 N. Young Ave., Fayetteville, Arkansas 72704, United States; 2 Research Institute for Raw Materials and Beverage Analysis, 234719 Versuchs- und Lehranstalt für Brauerei in Berlin (VLB) e.V., Seestr. 13, Berlin 13353, Germany; 3 Pabst Brewing Company, 711 Broadway, Suite 600, San Antonio, Texas 78215, United States; 4 International Centre for Brewing and Distilling, Institute of Biological Chemistry, Biophysics, and Bioengineering, School of Engineering and Physical Sciences, Heriot-Watt University, Edinburgh EH14 4AS, Scotland

## Abstract

Producing high-quality nonalcoholic beer (NAB) requires precise management of ethanol levels while maintaining desirable sensory properties. This study investigated the influence of high-gravity brewing (HGB), yeast strain selection, and glucose oxidase (commercially sold by dsm-firmenich as TasteZyme G (TG)) treatment on NAB quality. Fermentations were performed using two maltose-negative*Saccharomyces cerevisiae*strains (LalBrew LoNa and SafBrew LA-01) and an arrested fermentation with SafLager W-34/70 (*Saccharomyces pastorianus*) in 16.2–16.9% w/w HGB wort. Key parameters, including fermentation kinetics, ethanol formation, residual sugars, gluconic acid accumulation, volatile aroma compounds, and sensory characteristics (descriptive analyses and triangle tests), were assessed. LA-01 and LoNa produced beers with ethanol concentrations up to 1.53% v/v prior to dilution, while W-34/70 reached 0.78% v/v. TG treatment effectively decreased the glucose content and increased gluconic acid levels; however, after dilution, the impact on most NABs was minimal. Notably, TG addition reduced phenolic off-flavors associated with the POF^+^ LA-01 strain, while the effect on other products was not as clear. Sensory analysis revealed that NABs from maltose-negative strains exhibited a clean, balanced flavor profile, whereas W-34/70 samples were perceived as sweeter and more worty. The combined approach of HGB, strategic yeast selection, and enzymatic glucose modulation enabled precise control over ethanol, residual sugar, and aroma composition. These findings demonstrate that integrating fermentation management with targeted enzymatic treatment offers a practical strategy for producing NABs with low ethanol (<0.5% v/v), moderated sweetness, and enhanced aromatic complexity, supporting industrial-scale applications.

## Graphical abstract

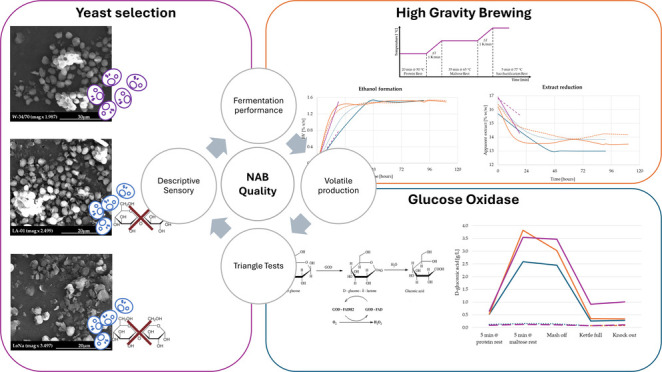

## 1 Introduction

Shifting lifestyle choices that emphasize health and mindful alcohol consumption have driven increasing consumer demand for low- and nonalcoholic beers (NABs).^1^ These beverages are generally defined as containing less than 0.5% alcohol by volume (ABV), with some products being entirely alcohol-free (<0.05% ABV).^2,3^ Their growing popularity is supported by benefits, such as isotonic properties aiding postexercise recovery, evolving societal attitudes toward alcohol consumption, stricter drunk-driving regulations, and diversification of consumer palates.^4^ From a technological perspective, NABs are produced through two main strategies: (1) biological methods, which suppress ethanol formation during fermentation, and (2) physical methods, which remove ethanol after a complete fermentation.^4–8^ Biological approaches involve adjusting wort composition, fermentation conditions, or employing specialized yeast strains to limit alcohol production,^9–13^ while physical methods include vacuum distillation or membrane-based separation techniques.^4,5,12,14,15^ Increasingly, hybrid approaches combine both strategies to better replicate the sensory complexity of full-strength beers.^6,7,16^

Despite these advances, NABs' chemical and sensory profiles can differ markedly from alcoholic beers.^17,18^ In general, NABs tend to contain lower levels of fermentation-derived aroma compounds such as higher alcohols, acetate esters, or ethyl esters, while certain aldehydes, typically associated with stale flavors in full-strength beers (beers with ABV concentrations >0.5%), are frequently present at elevated levels even in freshly produced NABs.^17,19^ A particular sensory challenge is the prevalence of “worty” flavors, primarily linked to Strecker aldehydes such as methional (3-(methylsulfanyl)­propanal) and/or 3-methylbutanal.^2,20,21^ These compounds occur in the microgram-per-liter range and strongly affect flavor quality.^22,23^ Strategies to mitigate these off-flavors include the use of alternative, nonmalted grains^20^ and/or downstream treatments such as adsorption techniques.^23^ Thus, NAB production represents not only a response to shifting consumer demand but also a complex technological and sensory challenge that continues to drive innovation in brewing science.

One technological strategy that offers potential benefits for NAB production is high-gravity brewing (HGB), which has been widely used in full-strength beer production.^24,25^ In this process, a concentrated wort is produced and the beer produced is later diluted/blended to the desired sales ABV concentration.^24,25^ Worts at 13–18% w/w (°Plato) are generally considered high-gravity, while very high-gravity brewing refers to worts above 18% w/w.^25,26^ The global relevance of HGB is considerable, and it is estimated that ∼80% of all beer produced is made with this type of methodology.^27,28^ HGB offers clear advantages such as smaller wort volumes requiring heating and cooling, reducing energy consumption, while blending produces larger final beer volumes, increasing plant throughput and product consistency through blending. These efficiencies translate into lower operating costs, water use, wastewater generation, and CO_2_ emissions, making HGB highly attractive in the context of sustainability goals and environmental impact reduction.^29^

However, the use of elevated wort concentrations also introduces technological challenges. Higher gravity worts demand optimized mash mixing and efficient liquid–solid separation, with mash filters generally outperforming traditional lauter tuns.^29^ In addition, elevated gravity can reduce hop isomerization and reduce foam stability (head retention) in the final diluted beer.^25,29,30^ Fermentation under these conditions places significant stress on conventional yeast strains, often leading to the increase in fermentation byproducts such as higher alcohols and esters, which can alter the sensory profile of full-strength beers.^25,29,31^ While this may be undesirable in regular beer production, it could be advantageous for NABs, which frequently lack sufficient concentrations of fermentation-derived flavor compounds. Careful yeast selection is therefore essential to harnessing the benefits of HGB in NAB production, combining technological efficiency with improved flavor characteristics.

Yeast strain selection has emerged as a key factor in biological NAB production.^9,10^ Traditional biological methods, such as employing elevated mashing temperatures (hot mash >75 °C) to reduce fermentable sugar formation and/or conducting cold-contact fermentations to limit yeast activity, have long been used to restrict alcohol formation.^21^ While effective at lowering ethanol levels, these approaches often result in beers with undesirable sensory characteristics, including excessive sweetness, wort-like flavors, and/or a lack of aroma complexity due to reduced formation of higher alcohols and esters.^9,10,19,20^ To overcome these drawbacks, researchers and brewers are increasingly investigating the use of nontraditional brewing yeasts.^9,10,32,33^ These strains can be integrated into existing brewery infrastructure and batch fermentation processes, making them more practical than continuous cold fermentation or physical dealcoholization methods. Such yeasts are sourced through bioprospecting, hybridization, or genetic modification.^9,10,13^ Their defining feature is the inability to efficiently metabolize the main fermentable wort sugars, particularly maltose and maltotriose.^13,20^ Instead, they primarily consume simple sugars such as glucose and fructose, which limits ethanol formation during fermentation.^10,20^ In moderate-strength worts, this restricted metabolism typically keeps ethanol concentrations below 0.5% ABV. At the same time, these yeasts produce secondary metabolites, including higher alcohols and esters, which contribute desirable beer-like aroma characteristics and facilitate the reduction of aldehydes responsible for “worty” flavors. Consequently, yeast selection and fermentation management are critical for producing NABs that are both technologically feasible and sensorially appealing.^20,34^

In addition to specialized yeasts, novel enzymatic tools further expand the possibilities of NAB production. One such innovation is glucose oxidase (TG). While used in the food industry (e.g., baking) or the production of low-alcohol wine for decades,^35–38^ it was first introduced commercially to the brewing industry by dsm-firmenich under the name Brewers TasteZyme G.^39^ TG is a flavoprotein enzyme (EC 1.1.3.4, β-d-glucose:oxygen 1-oxidoreductase) that catalyzes the oxidation of β-d-glucose to d-glucono-δ-lactone, with molecular oxygen serving as the electron acceptor and hydrogen peroxide (H_2_O_2_) produced as a byproduct. In the second step of the reaction, d-glucono-δ-lactone is converted into gluconic acid (eq 1):^36,37,39,40^

$$\beta ‐D‐\mathrm{glucose}+O_{2}\to D‐\mathrm{glucono}‐\delta ‐\mathrm{lactone}+H_{2}O_{2}\to D‐\mathrm{gluconic}\;\mathrm{acid}$$ 1

This reaction has some interesting implications for NAB brewing.^35–37,39^ First, the conversion of glucose to gluconic acid (GA) reduces the availability of simple sugars, thereby limiting ethanol formation, which is particularly relevant in the production of NABs. Second, increasing the amount of organic acid build can help to balance sweet-sour impressions in the final product. Finally, by consuming molecular oxygen, TG functions as an oxygen scavenger, mitigating the negative effects of hot-side aeration during mashing.^35–37,39^ Oxygen uptake during beer production is a major contributor to staling, as it oxidizes wort components and leads to undesirable color changes and stale flavors.^41^ By reducing oxygen levels early in the process, TG has the potential to improve both the flavor stability and shelf-life of NABs.

Taken together, the integration of high-gravity brewing, specialized yeast strains, and innovative enzymatic tools such as glucose oxidase represents a promising strategy for producing nonalcoholic beers that meet modern consumer expectations for sensory quality, technological efficiency, and sustainability. This study aimed to evaluate strategies for improving the production and sensory quality of NAB by combining yeast strain selection, HGB, and TG applications. Three yeast strains were investigated: two maltose-negative strains (SafBrew LA-01 and LalBrew LoNa) and *Saccharomyces pastorianus* (SafLager W-34/70) in an arrested fermentation approach. HGB was applied at 16–17% w/w to enhance ester formation, fruity aroma, and mouthfeel while increasing production efficiency in NAB brewing. To address the common issue of excessive sweetness in NABs caused by incomplete fermentation, TG was used to convert glucose into GA, introducing a natural acidity that balanced the flavor profile. Sensory and chemical analyses were conducted to assess the combined effects of HGB and TG treatment, with the goal of developing NABs with improved aroma, body, and drinkability through optimized fermentation management.

## 2 Materials and Methods

### 2.1 Wort and Beer Production

The following experimental design (Table 1) was developed to assess the individual and interactive effects of yeast strain, fermentation method, and glucose oxidase addition on the physicochemical properties and sensory acceptance of NABs produced using a high-gravity wort stream. The input parameters were selected to reflect commercially relevant options and practices applicable to both macro- and craft-scale breweries. Many brewing parameters were chosen to ensure the process compatibility and scalability for commercial production. The NABs in this study were brewed, fermented, filtered, and analyzed at the Center for Beverage Innovation, Department of Food Science, University of Arkansas (Fayetteville, AR, USA). NAB production was carried out using a single batch of wort produced under varying conditions (Table 1).

**Table 1:** Experimental Design

treatment	manuscript code	commercial name	fermentation @ 22.2 °C	glucose oxidase
LoNa	LoNa	LalBrew LoNa	to final gravity	none
LA-01	LA-01	SafBrew LA-01	to final gravity	none
W-34/70	AF	SafLager W-34/70	for 18 h	none
LoNa+TG	LoNa+TG	LalBrew LoNa	to final gravity	2 kg/T malt
LA-01+TG	LA-01+TG	SafBrew LA-01	to final gravity	2 kg/T malt
W-34/70+TG	AF+TG	SafLager W-34/70	for 18 h	2 kg/T malt

### 2.2 Wort Production

A high-gravity wort targeting 16.5% w/w (±0.5% w/w) was produced at a fermenter volume of 40 L using a 2-vessel recirculating pilot brewery system (mash and heating vessel, Ss Brewtech, Wildomar, CA, USA). The grist consisted of 74.00% 2-row malted barley (RahrBSG, Shakopee, MN, USA), 0.41% black 2-row malt (Briess Malt & Ingredients Co., Chilton, WI, USA), and 25.59% Brewers Crystals (GLOBE 55 HM Corn Syrup/Glucose Solids, Ingredion, Westchester, IL, USA). CaCl_2_ (RahrBSG) was added to achieve 113.2 mg/L calcium in the mash, and lactic acid (88%, LD Carlson Company, Kent, USA) was used to adjust the mash pH to 5.3 ± 0.1. Three worts received a glucose oxidase treatment (TasteZyme G, dsm-firmenich, Maastricht, Netherlands) at 2 kg/T of malt postmash-in; three others served as untreated controls. Mashing was performed (Figure 1), and saccharification was confirmed via iodine testing (0.02 N iodine, Merck KGaA, Darmstadt, Germany). Lautering with continuous sparging yielded 45 L of wort, which was boiled for 60 min with four hop varieties (Chinook, Nugget, Mt. Hood, and Willamette; BSG Hops, Wapato, WA, USA) at two addition times: 30 min (16.7 g of Chinook, 17.9 g of Nugget) and 10 min (12.9 g of Mt. Hood, 20.9 g of Willamette) to target 7.5 ± 0.5 BU in the final diluted NAB. Postboiling, wort underwent a 5 min whirlpool and 10 min settling to form trub. Cooling to 21 °C was performed via a single-stage plate heat exchanger with sterile oxygen injected at 0.12 L/min prior to pitching.

**Figure 1:**
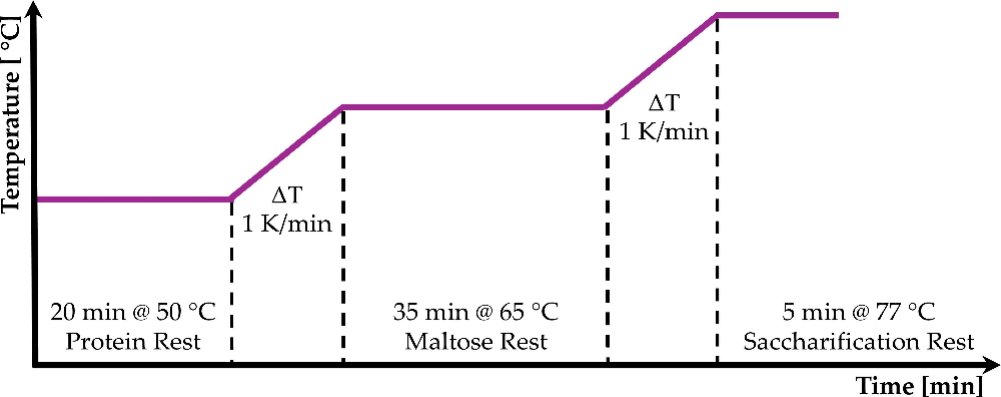
Mashing profile for NAB wort production.

### 2.3 Fermentation

Three commercially available dry brewing yeast strains were used for fermentation: two commercially marketed maltose-negative *Saccharomyces* strains, SafBrew LA-01 ^33^ and LalBrew LoNa,^32^ and a commonly used bottom-fermenting lager strain (*S. pastorianus*), SafLager W-34/70.^42^ The dry yeasts were directly pitched at 75 g/hL for LA-01 and LoNa and 100 g/hL for W-34/70. While the maltose-negative strains were pitched at the higher end (or above) of their recommended range, the pitching rate for the bottom-fermenting W-34/70 was set within the midrange of the manufacturer’s recommendations^32,33,42^ to ensure comparable conditions for these specific nonalchoholic HGB fermentation trials.

Fermentation was carried out in stainless-steel cylindroconical fermenters with a net volume of 53 L, maintained at a target fermentation temperature of 22.2 °C (±0.3 °C). To prevent the formation of diacetyl (butane-2,3-dione) and associated off-flavors, alpha-acetolactate decarboxylase (ALDC, Murphy & Son Limited, Nottingham, England) was added at a dosage of 1 mL/hL per fermenter.

The fermentations involving LalBrew LoNa and SafBrew LA-01 were allowed to proceed to terminal gravity, while those conducted with SafLager W-34/70 were arrested after 18 ± 1 h to limit alcohol formation and maintain similar fermentation times among both arrested products.

### 2.4 Downstream Processing (Filtration, Blending, Kegging, and Pasteurization)

Following fermentation, the conical fermenters were cooled to 2.4 °C to cold crash the fermentation. This facilitated yeast removal and further enhanced the clarification via sedimentation. The beers were transferred into 19.5 L stainless-steel buffer kegs to enable filtration at a higher pressure (maximum 2 bar, compared to the fermenters’ safety limit of 1.3 bar). Filtration was carried out using FIBRAFIX AF depth filter media (Filtrox AG, St. Gallen, Switzerland). The AF ST 110 filter sheets, with a nominal retention range of 0.8–0.5 μm (classified as germ-reducing), were installed in a Hobracol 200 Mikro filter (Hobra–Školník s.r.o., Broumov, Czech Republic). The setup consisted of a single inlet, two collecting frames (each with one outlet), and two filter sheets, providing a total filtration area of 0.064 m^2^. Prior to use, the filter was assembled, tightened, and rinsed with 90 °C water to remove loose particles, promote filter sheet swelling, and sterilize the system. The hot water was held in the filter for 10 min to ensure complete swelling and then flushed with sterile, deaerated water at 4 °C to cool the system.

The yeast-reduced NABs (kegs) were then connected to the filtration system and processed under a maximum pressure of 2 bar by using food-grade CO_2_. The first 2 L of filtrate was discarded to prevent dilution from residual water used during filter preparation. The clarified beer was collected into 19.5 L final kegs. The filtered HGBs were diluted with deaerated, sterile water to an ABV of 0.38%. The arrested fermentation sample without enzyme treatment yielded a lower final ABV of 0.21% due to its reduced fermentation activity. After dilution, the products were adjusted to a target pH of 3.95 ± 0.1 using food-grade phosphoric acid (85%, Alliance Chemical, TX, USA). Finally, the beers were force-carbonated with food-grade CO_2_ to a carbonation level of 5.4 g/L and pasteurized to a target of 25 pasteurization units (PU).

### 2.5 Chemical Analysis

#### 2.5.1 Physicochemical Analysis

Beer analyses were conducted using standardized methods outlined by the European Brewery Convention (EBC): density, original gravity, apparent and real extract (EBC 9.4; EBC 9.43.2), and alcohol content (EBC 9.2.6),^43–45^ and ASBC standard methods Beer Method 9-pH (Hydrogen Ion Concentration), Beer Method 10 A-Color (applying the conversion specified in the method (EBC = SRM × 1.97) to convert SRM to EBC units), and Beer Method 23 A-Bitterness were utilized.^46–48^

For the quantification of glucose, maltose, and maltotriose, a high-performance liquid chromatography (HPLC) method was applied as published by Schubert et al.^20^ Analyses were conducted using a Waters Arc HPLC system (Waters, Milford, MA, USA) equipped with a Waters Sample Manager, Quaternary Solvent Manager-R, and a column heater coupled to a Waters Acquity QDA detector.

Separation was achieved using a Rezex ROA-Organic Acid H+ (8%) column (300 × 7.8 mm, 8 μm; Phenomenex, Torrance, CA, USA) maintained at 65 °C, protected by a SecurityGuard Carbo-H cartridge (4 × 3.0 mm ID; Phenomenex). The mobile phase consisted of 0.1% formic acid delivered at a flow rate of 0.4 mL/min, with a total run time of 40 min. The injection volume was 0.1 μL. Calibration curves for all sugars were generated across a concentration range of 50–1500 ng/μL.

The determination of gluconic acid was carried out using a Megazyme “d-gluconic acid/d-glucono-δ-lactone” assay kit (Megazyme Ltd., Bray, Co. Wicklow, Ireland), following the manufacturers’ recommended protocol.^49^

#### 2.5.2 Volatile Analysis

Volatile compound analysis was carried out using a Shimadzu GC-MS/MS system (GCMS-TQ8050 NX, Shimadzu Corporation, Kyo̅to, Japan) equipped with an AOC-6000 Plus autosampler. For solid-phase microextraction (SPME), a 50/30 μm DVB/CAR/PDMS fiber (Supelco, Bellefonte, PA, USA) was used. Samples were incubated at 65 °C for 10 min, followed by extraction under identical conditions for another 10 min. Subsequently, the fiber was thermally desorbed in splitless mode at 240 °C for 3 min. The inlet temperature was kept constant at 240 °C, and helium served as the carrier gas.

The GC oven program started at 35 °C with an initial hold of 5 min, followed by a temperature ramp to 100 °C at 5 °C/min, to 150 °C at 3 °C/min, to 160 °C at 8 °C/min, and finally to 250 °C at 25 °C/min with a final hold of 5 min, resulting in a total run time of 39.52 min. Separation was achieved using an HP-5MS UI column (30 m × 0.25 mm × 0.25 μm; Agilent J&W GC Columns, Santa Clara, CA, USA). The mass spectrometer source temperature was set to 200 °C, and the transfer line was maintained at 280 °C.

Before quantification, standards were analyzed in full scan mode (*m*/*z* 40–400), followed by product ion scans to determine optimal collision energies. These data points were used to develop a multiple reaction monitoring (MRM) method. Compounds were organized into 11 groups, each containing several compound-specific transitions, with a loop time of 0.0550 s per group. The detector was operated at 1.3 kV, with ion source and interface temperatures set to 200 and 280 °C, respectively. Other parameters, including the oven program and injection settings, were identical to those used for the full scan method.

For quantification, an internal standard-based calibration curve with 10 concentration levels (10–10,000 μg/L) was prepared using a mixture of 41 reference compounds (purity >95%, Sigma-Aldrich, St. Louis, MO, USA) covering various chemical classes, including esters, aldehydes, terpenes, and terpene alcohols. Calibration solutions contained 500 μg/L of five isotopically labeled internal standards (hexanal-*d*_12_, ethyl hexanoate-*d*_11_, phenylacetaldehyde-*d*_5_, linalool-*d*_5_, and β-myrcene-*d*_6_; purity >94%, AromaLAB GmbH, Planegg, Germany), each representing a different compound class. All calibration curves showed excellent linearity (*R* ^2^ > 0.999). Sample analytes were quantified based on the internal standard assigned to their respective compound class.

### 2.6 Sensory Analysis

#### 2.6.1 Triangle Tests Performed and Panel Description

Triangle tests were conducted in accordance with ISO 4120:2021.^50^ The sensory panel consisted of 15 participants (age 21–38 years; 11 male, 4 female). The sensory analyses were performed in a single session in March 2025. An α-risk level of 0.05 was selected, representing moderate evidence for detecting a perceptible difference.^50^ According to the corresponding statistical parameters, a minimum of nine correct identifications of the different samples in the forced-choice triangle test was required to establish statistical significance.

#### 2.6.2 Descriptive Sensory Performed and Panel Description

Descriptive analysis was conducted following established sensory evaluation methodologies as published.^51,52^ A trained panel consisting of two subgroups (both will be presented separately) characterized the sensory attributes of the six NABs produced in duplet. The two panels consist of (i) University of Arkansas: age 27–36, 3 male, 5 female; and (ii) commercial brewery panel: age 30–55, 6 male, 8 female.

Panelists were asked to smell (S) the samples first and rate the overall aroma intensity of the following characteristics: worty, cereal, hoppy, phenolic, and cheesy. Subsequently, panelists were asked to consume the NABs and rate the taste (T) impressions for their impressions of viscosity/mouthfeel, bitterness, sweetness, and sourness. As a final description, the panelists were asked to rate the retronasal (RN) impressions, overall intensity, worty, cereal, hoppy, phenolic, and cheesy.

The sensory analyses were performed over 1 week in March 2025. The panelists self-identified as beer consumers on a regular basis and were available to participate in both evaluation sessions. Ethical permission was evaluated, and the study protocol (IRB project no. 2309492758) was exempted by the institutional review board. All panelists gave their consent to participate in evaluating the samples.

### 2.7 Data Collection and Statistical Analysis

For data collection, Microsoft Excel (Microsoft 365, Microsoft Corporation, Redmond, Washington, USA) was used. The data analysis was performed using XLSTAT statistical and data analysis software (Lumivero, New York, USA).

## 3 Results and Discussion

### 3.1 Wort Production, Fermentation, and Basic NAB Results

One of the main objectives of this study was to evaluate the suitability of commercially marketed maltose-negative yeast strains (i.e., LoNa and LA-01) for nonalcoholic beer production under high-gravity brewing conditions. For comparison, a lager strain (i.e., W-34/70 (*S. pastorianus*)) was also included in this study. This yeast is widely used in conventional full-strength and HGB beer production but has also been adapted to NAB brewing under restricted fermentation conditions, such as reduced temperature (i.e., cold contact) or fermentation time.^42,53–55^

Worts were produced with a target original gravity (OG) of 16.5 ± 0.5% w/w (Table 2). Overall, these values substantially exceed the 6–8% w/w typically used in NAB brewing with maltose-negative yeasts,^11,13^ where the objective is to achieve an alcohol content of less than 0.5% v/v.^2^ The targeted OG not only represents high gravity in the context of NAB but also exceeds the OG strength of many conventional full-strength beers (i.e., ∼12% w/w). All trials reached the specification (OG 16.5 ± 0.5% w/w), except for the LoNa+TG brew, which achieved 15.7% w/w (Table 2). These results confirm that the requirement for high-gravity brewing was successfully met across all of the experimental trials.

**Table 2:** Basic Analytical Data of High-Gravity Wort (Fermenter-Full)^a^

treatment type	original gravity* [% w/w]	wort pH* [−]	glucose [g/L]	maltose [g/L]	maltotriose^$^ [g/L]
LoNa	16.4	5.17	14.4^a^ ± 0.12	140.1^a^ ± 0.34	39.2^ab^ ± 0.14
LA-01	16.4	5.13	15.3^a^ ± 0.48	140.8^a^ ± 2.46	40.3^a^ ± 0.16
AF	16.8	5.20	15.0^a^ ± 0.53	138.6^a^ ± 0.16	39.3^ab^ ± 0.09
LoNa+TG	15.7	5.08	14.8^a^ ± 0.18	132.5^b^ ± 1.48	38.1^b^ ± 0.01
LA-01+TG	16.2	4.98	14.4^a^ ± 0.10	138.2^a^ ± 1.39	39.3^ab^ ± 0.12
AF+TG	16.9	4.86	12.6^b^ ± 0.44	130.0^b^ ± 1.91	38.8^ab^ ± 1.05

a ±Variation expressed as standard errors. *Original gravity and pH measured in singlet. ^$^Nonsignificant ANOVA results. Superscript letters indicate LS mean groupings (Fisher LSD, *p* < 0.05).

Wort pH values (Table 2) were higher than those usually associated with NAB production. Fermenter-full worts showed pH values between 4.86 (AF+TG) and 5.20 (AF) (Table 2). Although not performed in this study, mash acidification below pH 4.8 is recommended to control potential spore formers. Conventional restricted and special yeast fermentations using worts with 6–8% w/w original gravity result in limited production of organic acids (∼0.3 pH reduction).^10,20^ Overall, since a pH < 4.2 is critical to limit the growth of food-borne pathogens, NABs with a final pH > 4.2 must be acidified. Therefore, downstream processes were implemented such as the dilution with degassed water, controlled acidification with food-grade phosphoric acid to adjust the final pH (3.95 ± 0.1), and pasteurization (25 PU). These steps ensured compliance with both safety and quality aspects, highlighting the importance of integrating downstream adjustments into high-gravity NAB brewing.

Carbohydrate analysis (Table 2) confirmed maltose as the predominant sugar fraction in all of the worts. This is notable as the applied yeast strains were chosen for their inability to metabolize polysaccharides with a degree of polymerization ≥2 (ex. maltose and maltotriose). Glc, Mal, and Mtr concentrations were broadly comparable among brews. Notably, AF+TG contained the lowest concentrations of glucose (12.6 g/L) and maltose (130.0 g/L), while maltotriose levels were also on the lower end (38.8 g/L). The overall sugar spectrum and carbohydrate ratios in the samples reflect the use of brewer crystals (e.g., 8% glucose, 55% maltose, and 17% maltotriose in dry matter).^56,57^ Collectively, the carbohydrate data confirm that the wort composition was balanced across the treatments and provided a consistent substrate for fermentation trials.

Glucose variations reflected the effect of enzymatic treatment. As expected, TG treatment reduced Glc concentrations due to conversion into d-gluconic acid. In agreement with past research,^38^ during mashing, the concentration of d-gluconic acid increased steadily, which was mirrored by the decline in glucose (Figure 2). The rise coincides with the start of the protein rest and reflects peak catalytic activity just before thermal inactivation (at 60 °C starting with the saccharification rest), which occurs rapidly at temperatures above 60 °C.^58,59^ These results confirm that the enzymatic conversion of Glc to GA proceeded as intended and that process conditions (such as temperature) strongly influenced the magnitude of the effect. Sparging during lautering diluted the kettle wort and reduced the concentration of d-gluconic acid (evident in the drop from mash-off to kettle-full).

**Figure 2:**
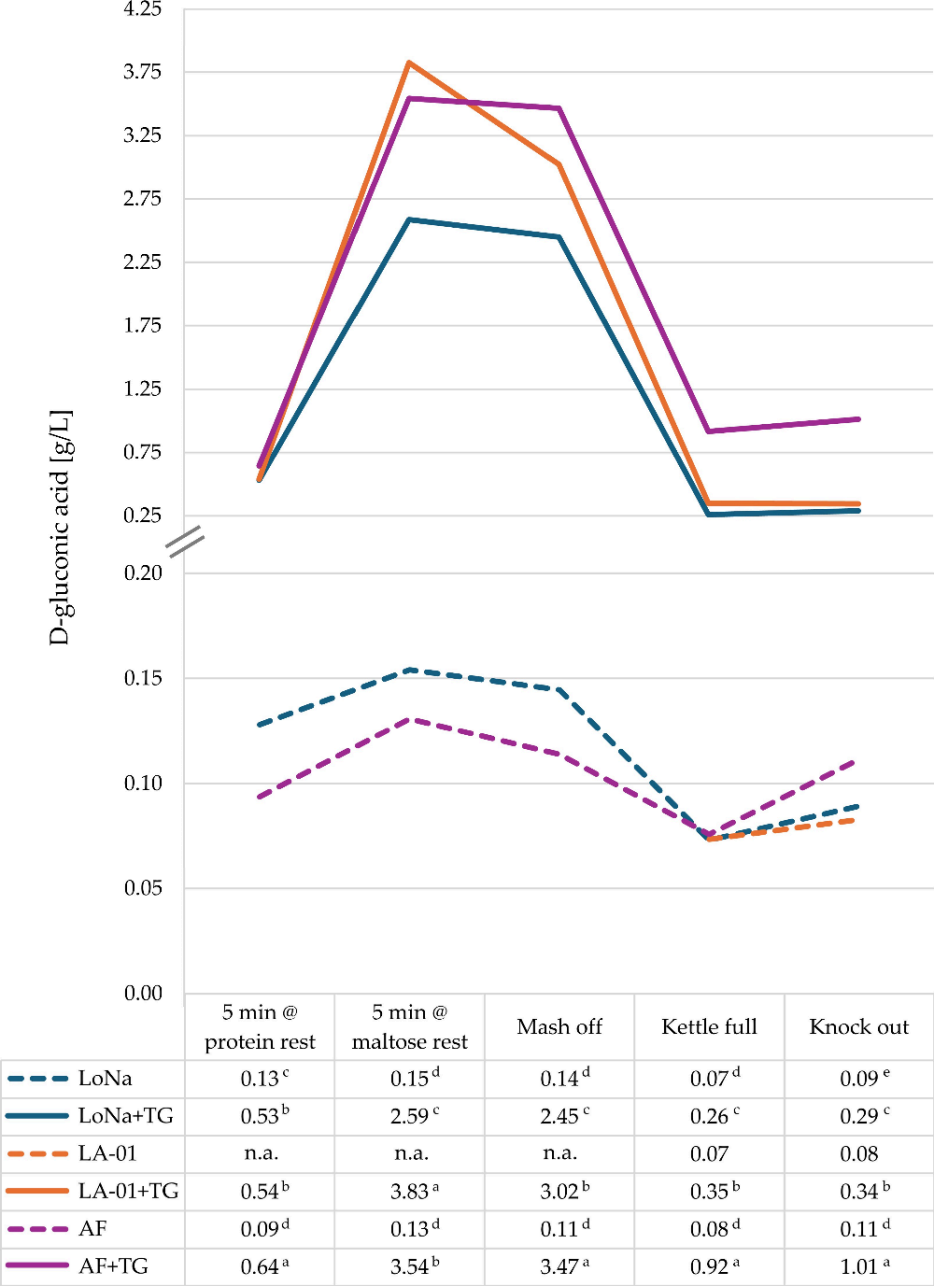
Gluconic acid (GA) concentrations during wort production from mashing to knockout. Superscript letters indicate LS mean groupings based on Fisher’s LSD test (*p* < 0.05). Missing values for yeast strain LA-01 result from missing sample volumes for GA analysis (n.a.). Please note the axis break on the *y*-axis between 0.20 and 0.25 g/L GA.

Beyond wort and enzyme characterization, the study evaluated the fermentation performance of commercial maltose-negative NAB yeast strains LoNa and LA-01, widely used in research and practice, with the lager strain W-34/70 (*S. pastorianus*) included as a benchmark.^9,10,34^ As ALDC was utilized to reduce diacetyl production, there was minimal need for conditioning, and fermentations were crash-cooled once the gravity had stabilized. The arrested fermentation (W-34/70) was stopped at 18 h (AF/AF+TG), whereas fermentations with LA-01/LA-01+TG completed after 110 h, and those with LoNa/LoNa+TG were completed in 91 h (Figure 3A). These differences in fermentation time between LA-01 and LoNa can be linked to the ability of S. *cerevisiae* var. *chevalieri* (LA-01) to partially ferment maltose (up to 25%^60^), which triggers a delayed (second) fermentation phase beginning after approximately 96 h.^10^ While the arrested fermentations reached a final ABV of 0.78%, the other treatments fermented to comparable final alcohol levels, ranging from 1.49% (LA-01+TG) to 1.53% (LA-01 and LoNa+TG) (Figure 3B). These results were expected and aligned with the original recipe formulation for treatments using SafBrew LA-01 and LalBrew LoNa, as both yeast strains are characterized by very low attenuation (SafBrew LA-01 15%; LalBrew LoNa 16–20% ^32,33^ ), which is consistent with previous findings in products with comparable original gravities (original extract 4.5% w/w: LoNa 13.26%, LA-01 22.49%; original extract 9% w/w: LoNa 13.30%, LA-01 17.45%).^10,34^ Previous research^61^ also showed that fermentations with LA-01 and LoNa in worts of 6.50% w/w original gravity reached an apparent degree of fermentation (ADF) comparable to that observed in the current study. This highlights the applicability of these strains in high-gravity NAB production, especially given that wort fermentability decreases as the original gravity increases. Overall, ADF values were higher for all samples receiving enzymatic treatment compared to those without TG treatment. For the AF set, the increase in ADF was substantial (ΔADF = 7.5% w/w), whereas the increases were smaller for the LA-01 (ΔADF = 0.5% w/w) and LoNa (ΔADF = 1.0% w/w) sets (Table 3). Since glucose oxidase converts glucose to gluconic acid,^40^ a reduction in fermentable sugars would be expected, potentially leading to lower ADF values. Instead, the increase in ADF may indicate a shift in sugar utilization dynamics, which might also influence the sensory properties of these NABs by reducing perceived sweetness and increasing perceived sourness. The pH data (Tables 2 and 3) support this observation. TG-treated samples consistently showed lower wort pH values, although this acidification effect was partially offset by organic acids produced by the yeast during fermentation. Elevated pH levels in untreated samples are a concern for food safety, particularly after product expansion, as pH values above 4.6 can support the growth of pathogenic microorganisms.^62,63^ To mitigate this risk, the final beers were adjusted to a safe pH of 3.95 ± 0.05 during dilution.

**Figure 3:**
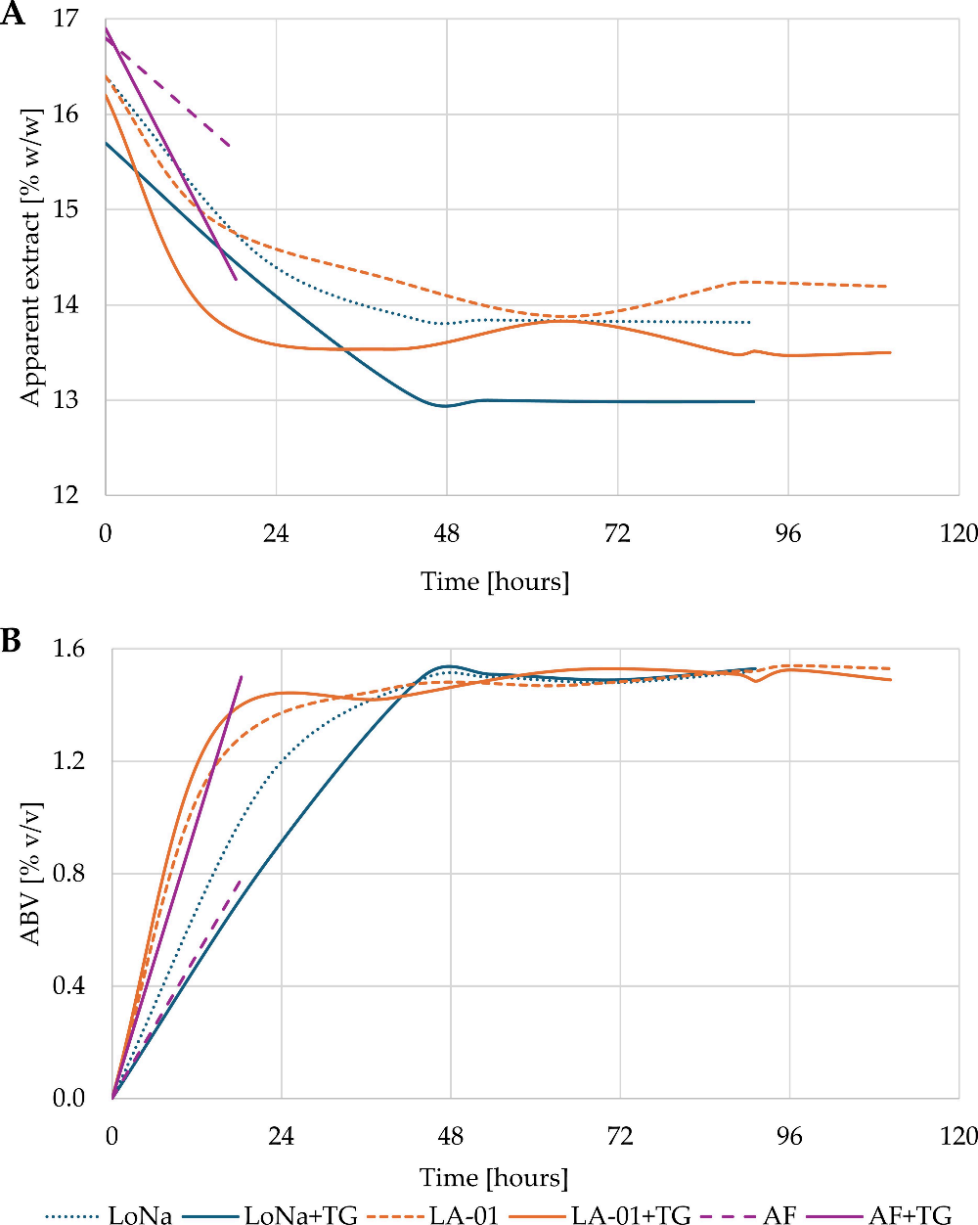
Fermentation performance across the different nonalcoholic beer production approaches with regard to (A) extract reduction and (B) ethanol formation.

**Table 3:** Basic Chemical Beer Results before and after Reduction (Dilution)^a^

		AF	AF+TG	LA-01	LA-01+TG	LoNa	LoNa+TG
before reduction	alcohol [% v/v]	0.78^b^ ± 0.03	1.50^a^ ± 0.01	1.53^a^ ± 0.00	1.49^a^ ± 0.00	1.52^a^ ± 0.00	1.53^a^ ± 0.00
	original gravity [% w/w]	17.0^a^ ± 0.05	16.9^a^ ± 0.00	16.9^a^ ± 0.04	16.2^c^ ± 0.00	16.5^b^ ± 0.00	15.7^d^ ± 0.01
	ADF [%]	8.1^e^ ± 0.29	15.6^d^ ± 0.09	15.9^cd^ ± 0.01	16.4^b^ ± 0.00	16.3^bc^ ± 0.00	17.3^a^ ± 0.00
	pH [-]	4.60^a^ ± 0.01	4.37^c^ ± 0.07	4.55^ab^ ± 0.01	4.51^ab^ ± 0.01	4.52^ab^ ± 0.00	4.50^b^ ± 0.01
after reduction	alcohol [%v/v]	0.22^c^ ± 0.00	0.44^a^ ± 0.02	0.42^b^ ± 0.00	0.41^b^ ± 0.00	0.43^ab^ ± 0.00	0.44^a^ ± 0.00
	original gravity [% w/w]	5.1^a^ ± 0.02	4.7^bc^ ± 0.10	4.9^a^ ± 0.04	4.6^c^ ± 0.02	4.9^ab^ ± 0.01	4.7^bc^ ± 0.08
	ADF [%]	8.3^d^ ± 0.05	18.0^a^ ± 0.04	16.6^c^ ± 0.19	17.3^b^ ± 0.02	17.2^b^ ± 0.03	18.2^a^ ± 0.28
	pH [-]	3.89^b^ ± 0.01	3.90^a^ ± 0.01	3.91^cd^ ± 0.03	3.97^ab^ ± 0.01	3.94^bc^ ± 0.01	3.98^a^ ± 0.01
	bitterness [BU]	7.2^b^ ± 0.10	7.9^a^ ± 0.23	7.1^b^ ± 0.20	7.0^b^ ± 0.00	7.0^b^ ± 0.35	6.5^b^ ± 0.05
	color [EBC]	4.8^a^ ± 0.06	4.5^b^ ± 0.02	4.3^c^ ± 0.02	4.4^b^ ± 0.05	3.9^d^ ± 0.02	4.2^c^ ± 0.03

a Superscript letters indicate LS mean groupings (Fisher LSD, *p* < 0.05).

The AF sample produced slightly more than half of the alcohol concentration observed in AF+TG and the other treatment groups. Because SafLager W-34/70 is a highly attenuating yeast strain with a comparable high alcohol tolerance in full-strength beer production (apparent attenuation 80–84%; alcohol tolerance 9–11%^42^), the arrested fermentation trials were deliberately stopped at 18 ± 1 h to achieve a maximum of 1.50% ABV. This higher ABV target was chosen to account for the planned product expansion (dilution) of 3.43, which would result in a final ABV below 0.5% (e.g., the legal US requirements for nonalcoholic beers (NABs)).^2,3^ Both the AF and AF+TG samples were stopped at the same time to maintain consistency, even though the AF sample had not yet reached the target ABV.

Examination of the basic HGB wort analysis (Table 2) suggests that the higher initial pH of the AF wort may have delayed yeast metabolism, potentially leading to an extended lag phase. Given that the total fermentation time was just beyond the typical lag phase window (3–15 h), a delayed onset of fermentation due to elevated pH could explain the lower ABV observed in AF. As expected, ethanol production (Figure 3) was directly correlated with extract decrease. The resulting apparent degree of fermentation (ADF), which reflects the fermentation performance and sugar utilization, ranged from 8.1% in the AF fermentation to 17.3% in the LoNa+TG trial (Table 3). Differences observed (Table 3; before reduction) can also be linked to the fact that the three yeast strains investigated originate from distinct *Saccharomyces* spp. (*S. cerevisiae* var. *chevalieri, S. cerevisiae,* and *S. pastorianus*). These taxonomic differences restrict not only the ability to metabolize carbohydrates (beyond maltose) but also genome-derived variances that influence fermentation performance. Each *Saccharomyces* spp. carries characteristic variations in regulatory pathways for sugar uptake (e.g., maltose), stress response, and nitrogen utilization, which in turn results in different fermentation kinetics. Recent findings by Maust et al.^10^ showed a new increase in alcohol formation (or the corresponding decrease in apparent extract) after a plateau at 96 h of fermentation with *Saccharomyces cerevisiae* var. *chevalieri*, which is also consistent with recent findings showing that this yeast still consumes about 25% of the available maltose.^60^ This maltose utilization could also explain the observed ADF values (Table 3) of 15.9% (LA-01) and 16.4% (LA-01+TG), which exceed the 15% reported by the producer.^33^

Based on a physicochemical perspective, the final products met all predefined specifications. All beers met legal requirements for U.S. nonalcoholic beer classification (<0.5% ABV^3^), bitterness levels were in the target range (7.5 ± 0.5 BU), and color values were consistent with expectations for a light, pale NAB (4.5 ± 0.5 EBC) (Table 3).

### 3.2 Volatile and Sensory Analysis

The sensory results presented in this study are based on fresh nonalcoholic beers served from 19.5 L stainless-steel kegs stored 2 weeks (4 °C) after downstream processing.

#### 3.2.1 Difference Testing: Triangle Tests

Triangle tests (between samples made with the same yeast with and without TG) were implemented to characterize whether the enzyme treatment was leading to a perceptible sensory difference. In accordance with ISO 4120:2021,^50^ a significance level of α = 0.05 was selected for the evaluation of the triangle tests (Table 4). With a panel size of 15 participants, a minimum of nine correct responses was required to achieve statistically significant detection of a difference.^50^

**Table 4:** NAB Sensory–Triangle Test Performed at the University of Arkansas

samples tested	panelists detecting the difference (correct answers)	statistical relevance 9 out of 15 (α = 0.05)
AF and AF+TG	8 of 15	no difference detected
LA-01 and LA-01+TG	11 of 15	difference detected
LoNa and LoNa+TG	7 of 15	no difference detected

Overall, the panel did not detect any significant differences between beers brewed with or without TG either when LoNa yeast was used or when arrested fermentation was applied (Table 4). The only treatment for which a significant difference was detected was the LA-01 fermentation. Given that the triangle test provides only a binary output (“difference detected” or “no difference detected”) without revealing the origin or nature of any difference, descriptive analysis was explored further.

#### 3.2.2 Sensory Profiles and Volatile Composition of NABs

For descriptive sensory analysis, the products were evaluated randomly across three sessions by two trained panels (i.e., at the University of Arkansas (UA) and at a commercial brewery (CB)). Analysis of variance followed by Fisher’s least-means-squared groupings was used to process the data, and only sensory terms differing significantly among the samples (α = 0.05) were considered for further analysis.

In agreement with the triangle test results, both sensory panels identified clear phenolic impressions in the LA-01 samples (Figure 4). The sample not treated with TG had the highest phenolic ratings across both the orthonasal and retronasal evaluations. LA-01 (*Saccharomyces cerevisiae* var. *chevalieri*) is a phenolic off-flavor (POF^+^) yeast (i.e., produces spicy, clove-like, or medicinal flavors by converting ferulic acid into 4-vinylguaiacol (4VG)).^10^ Previous research has demonstrated that pH adjustments during fermentation with POF^+^ yeasts can substantially reduce phenolic off-flavor production.^64^ For instance, lowering the wort pH to 4.2 using lactic acid achieved a 19% reduction in 4-vinylguaiacol (4VG), while kettle souring with *Lactiplantibacillus plantarum* prior to fermentation resulted in a 96% reduction. This effect has been linked to the activity of two key enzymes: phenylacrylic acid decarboxylase (PAD1) and ferulic acid decarboxylase (FDC).^65^ These enzymes decarboxylate phenolic precursors such as cinnamic acid and ferulic acid, producing 4VG and related compounds. Their enzymatic activity is optimal at pH 7.0–8.0 but declines sharply under acidic conditions.^65^ Although the pH of mash, wort, and especially beer is typically below 5.5,^4,8^ it is plausible that intracellular pH influences PAD1 and FDC activity, thereby modulating phenolic expression. Consequently, lowering the pH during fermentation could partially suppress the POF^+^ pathway, leading to decreased phenolic flavor formation. This aligns with the observed decrease in phenolic intensity when LA-01 was treated with TG, which produces gluconic acid^38,40^ and lowers the pH of the wort to be fermented.

**Figure 4:**
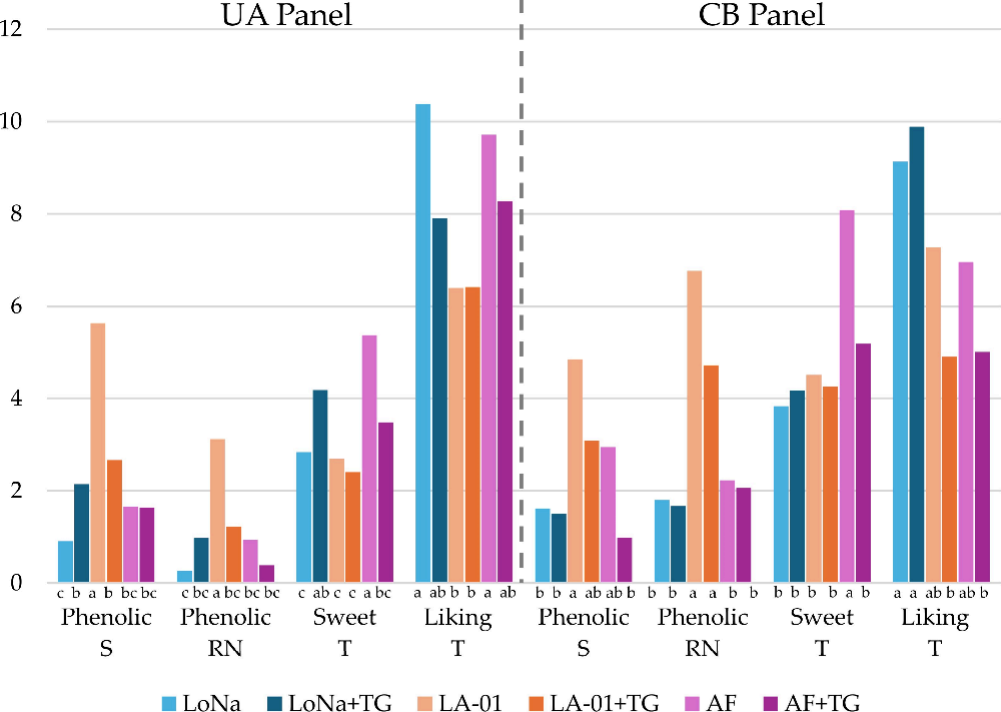
Selected ANOVA-based sensory ratings of the panel of University of Arkansas (UA) and the panel of commercial brewery (CB). Sensory results labeled S correspond to smell, T to taste, and RN to retronasal sensory ratings, while letters in lower case indicate LS mean groupings (Fisher LSD, *p* < 0.05).

A principal component analysis (PCA) was performed to investigate relationships between sensory attributes, chemical composition, and treatment effects across all six nonalcoholic beers (NABs). The first two principal components explained 61.86% of the total variance (*F*1 = 36.91%, *F*2 = 24.95%) (Figure 5A), while *F*3 accounted for an additional 17.52% of the variance (Figure 5B).

**Figure 5:**
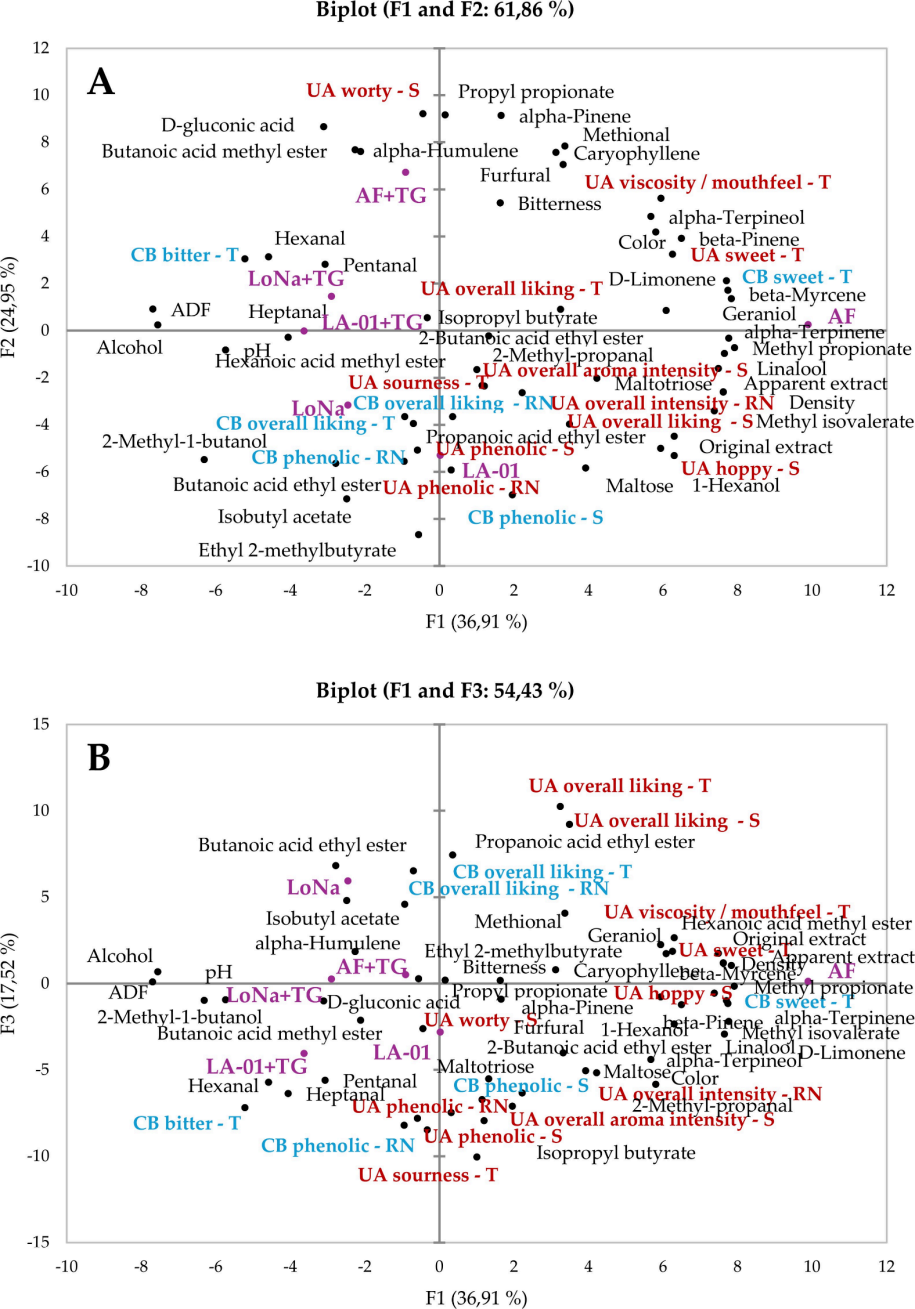
PCA plots showing the correlations between the UA panel (red) and the CB panel (blue) and the significant analytical results (black) in all NABs produced (purple). Sensory results labeled S correspond to smell, T to taste, and RN to retronasal sensory ratings. PCA “A” presents the principal components F1 and F2, showing the highest variance (61.86%), while PCA “B” presents the principal components F1 and F3, adding further information (total variance explained: 54.43%) by illustrating an additional level of correlation. Significant results after performing ANOVA were used as input variables only.

PC1 primarily separated the samples by the apparent degree of fermentation (ADF), residual sugars, sweetness, and bitterness. The AF sample was positioned on the far right of PC1, characterized by high residual sugars (apparent extract, maltose, and maltotriose), sweetness ratings, and elevated hop-derived volatiles such as linalool and geraniol. These characteristics reflect the low apparent degree of fermentation and lowest alcohol content created by the AF treatment (Table 3). In contrast, the LoNa and LoNa+TG samples were positioned on the left side of PC1 associated with higher ADF values (16.6–18.0%), compared to AF, which had an ADF of only 8.3%. The higher ADF was associated with lower residual sugars and, consequently, higher ABV levels. TG treatment of the LoNa sample caused only a slight positional shift, indicating a relatively minor effect when these high-gravity beers were diluted to their final strength.

PC2 was primarily driven by phenolic attributes and aldehyde compounds. The untreated LA-01 sample clustered strongly at the lower end of PC2, correlated with high phenolic aroma and retronasal scores. These findings are consistent with the significant differences identified by both triangle tests and ANOVA analyses (Table 4 and Figure 4). LA-01 exhibited a distinct and intense aroma profile, but its high phenolic character introduced polarizing sensory attributes, as reflected in its lower overall liking scores (Figure 4). The TG-treated variant (LA-01+TG) shifted upward on PC2, indicating a reduction in phenolic intensity and a movement away from residual sugar concentrations. This corresponded to lower bitterness ratings and a milder, more balanced aroma.

Along PC1, samples with sensory attributes, including sweetness, mouthfeel, and hop aroma, clustered on the right side of the biplot, particularly the AF treatment (Figure 5A,B). These attributes were closely associated with higher liking scores from the UA panel, suggesting that trained panelists preferred beers with these characteristics (Figure 5A). Conversely, samples on the left side of PC1 were associated with higher bitterness and lower sweetness, aligning with the analytical data for ADF and sugar concentrations.

TG treatment consistently caused samples to shift away from sweet and worty characteristics and toward increased sourness and reduced retronasal phenolic expression, most prominently in the LA-01 samples (Figure 5B). This effect is consistent with the known biochemical action of TG,^37,38,40^ which produces gluconic acid and thereby alters the flavor balance by reducing sweetness while increasing perceived acidity. These results highlight the potential of TG as a tool for modulating both chemical composition and sensory attributes, although its impact appears to depend on the yeast strain and initial wort composition.

Overall, the integration of sensory and analytical data demonstrates that both yeast strain and enzymatic treatment significantly influence the final quality of the NABs produced under HG conditions. LA-01 was primarily differentiated by its strong phenolic expression, while AF was distinguished by residual sugars and hop volatiles. LoNa and LoNa+TG clustered closely, showing that TG treatment exerted only a minor effect on HGB. Future studies should investigate the impact of applying TG throughout fermentation to determine how timing and dosing of TG may influence residual glucose levels and resulting sensory perception.

## 4 Conclusions

This study demonstrated that HGB, yeast strain selection, and enzymatic glucose modulation could effectively be combined to produce NABs with a controlled ethanol content, desirable sensory properties, and consistent physicochemical characteristics. Worts produced for this study reached the targeted high original gravity (16.2–16.9% w/w), significantly exceeding typical NAB formulations (6–8% w/w),^11,13^ thereby demonstrating that maltose-negative and restricted fermentation yeast strains can perform under high-gravity conditions. Carbohydrate analyses confirmed maltose as the predominant sugar fraction, consistent with limited metabolization by the applied maltose-negative strains (LoNa and LA-01) and the arrested fermentation setup with W-34/70. This restriction successfully limited ethanol formation, achieving ABV levels below 0.5% after dilution while maintaining balanced wort composition across treatments.

Enzymatic treatment with TG effectively reduced glucose concentrations through conversion into gluconic acid,^37,38,40^ demonstrating that targeted enzymatic interventions can modulate wort composition and downstream fermentation dynamics. Surprisingly, ADF increased in TG-treated samples despite a reduced glucose content, indicating potential shifts in sugar utilization patterns. These shifts contributed to altered sensory perceptions, particularly reduced sweetness and enhanced acidity, highlighting the interplay between carbohydrate composition, yeast metabolism, and enzymatic activity in shaping final beer flavor.

Fermentation performance differed between yeast strains: maltose-negative strains (LoNa and LA-01) completed fermentation over 91–110 h, producing slightly higher ethanol levels predilution compared to the arrested W-34/70 fermentation (0.78% v/v). Differences in ABV and ADF across treatments reflected both yeast-specific attenuation capacities and the influence of initial wort pH, which was higher than conventional NAB ranges but mitigated by controlled downstream acidification to ensure microbial stability (pH < 4.2) and sensory balance.

Sensory analysis revealed distinct strain-dependent profiles. LA-01, a POF^+^ strain, exhibited strong phenolic aromas, which were partially mitigated by TG treatment through a pH reduction and gluconic acid production during mashing. LoNa and W-34/70 samples were characterized by an absence of phenolic notes and instead showed subtle worty and fermentation-related characteristics, with only minor effects observed from TG addition. Principal component analysis confirmed that the fermentation degree, residual sugars, bitterness, and phenolic intensity were the main drivers of sensory differentiation among the treatments and demonstrates that both yeast selection and enzymatic intervention can be strategically applied to tailor specific flavor attributes, including sweetness, sourness, and phenolic character.

Overall, this study provides a comprehensive framework for industrial NAB production, demonstrating that high-gravity wort, specialized maltose-negative yeasts, and enzymatic glucose modulation can be combined to produce beers with low ethanol (<0.5% v/v), controlled residual sugars, and targeted aromatic profiles. These findings underscore the potential for fine-tuning fermentation management and enzymatic treatment to achieve reproducible, high-quality NABs, while offering practical guidance for optimizing sensory outcomes in large-scale brewing operations.

## Data Availability

The original contributions presented in the study are included in the article; further inquiries can be directed to the corresponding authors.
